# Blood, meat, and upscaling tissue engineering: Promises, anticipated markets, and performativity in the biomedical and agri-food sectors

**DOI:** 10.1057/s41292-017-0072-1

**Published:** 2018-01-15

**Authors:** Neil Stephens, Emma King, Catherine Lyall

**Affiliations:** a Social Sciences and Communications, Brunel University London, Kingston Lane, Uxbridge, Middlesex 01895268460, UK; b University of Stirling, Stirling, UK; c University of Edinburgh, Edinburgh, UK

**Keywords:** cultured blood, cultured meat, in vitro meat, promise, anticipated markets, tissue engineering

## Abstract

Tissue engineering is a set of biomedical technologies, including stem cell science, which seek to grow biological tissue for a diversity of applications. In this paper, we explore two emergent tissue engineering technologies that seek to cause a step change in the upscaling capacity of cell growth: cultured blood and cultured meat. Cultured blood technology seeks to replace blood transfusion with a safe and affordable bioengineered replacement. Cultured meat technology seeks to replace livestock-based food production with meat produced in a bioreactor. Importantly, cultured meat technology straddles the industrial contexts of biomedicine and agrifood. In this paper, we articulate (i) the shared and divergent promissory trajectories of the two technologies and (ii) the anticipated market, consumer, and regulatory contexts of each. Our analysis concludes by discussing how the sectoral ontologies of biomedicine and agri-food impact the performative capacity of each technology’s promissory trajectory.

## Introduction

Tissue engineering, and the related scientific fields of stem cell science and regenerative medicine, involves working to control the growth capacity of cells to produce useful tissue, typically for medical contexts. The aim is to replace damaged tissue (e.g., retinas or insulinproducing cells) or to stimulate healthy cell growth with the recipient’s own cells (e.g., kidney tissue). Alternatively, engineered tissue could be produced for testing and developing the effectiveness or the side effects of drugs within the laboratory. Typically, tissue engineering research is small scale and conducted in petri dishes. A small number of tissue engineering technologies are, however, targeting a step change in the upscale of stem cellbased work that would see a significant increase in productive capacity.

In this paper, we compare the promissory narratives and imagined market contexts of two of these innovative technologies: cultured blood and cultured meat. Both are among a group of technologies exploring the upscale of tissue engineering through mass culturing in bioreactors: sterile ‘vats’ that control for temperature and gaseous content that encourage cell growth in liquid media. Cultured blood and cultured meat are not the only tissue engineering technologies entering this space, but they are by far the two technologies with the most ambitious targets for the quantities of tissue produced with, ultimately, cultured blood seeking to replace all blood donation and cultured meat seeking to replace all meat consumption. The quantities involved here would truly constitute a step change in the upscale capacity if delivered, with, for the UK alone, total annual blood use being around 176 metric tonnes, and total annual meat consumption over five millions tonnes.^1^ However, considerable technical challenges remain. Cultured blood and cultured meat are different to existing cell bioreactor work, for example vaccines, that expand cells to collect what they secrete because, for cultured blood and cultured meat, the final products are the cells themselves (Hodson, 2015). Other uncertainties exist, for example for cultured meat whether the cells really will change from being endothermic (requiring additional heat) to exothermic (creating their own heat) as theoretically predicted, and to what degree. Subsequently, both technologies are early stage, fitting Hedgecoe’s (2004) notion of “promissory science” with more by way of future orientated projections than tangible product.

Cultured blood and cultured meat are good case studies to compare because of the distinctiveness of some of their shared characteristics (most notably in terms of upscale) and their key differences (especially their contrasting market contexts). Both cultured blood and cultured meat involve producing tissue intended to enter the human body, although their method of entry is quite distinct. For cultured blood, entry would be intravenous with subsequent flow through the cardiovascular system. For cultured meat, in contrast, the tissue would enter the body orally and pass through the digestive system. Beyond this, the social context of entry also differs, with cultured blood entering the body in contexts of licensed biomedical intervention and cultured meat via home cooking, restaurants, and canteens. Our account is divided into six sections: the first articulates the wider context of cultured blood and cultured meat; the second describes our methods; the third introduces our theoretical approach; the fourth compares the promissory narratives of cultured blood and cultured meat; the fifth articulates the anticipated market conditions of proponents in each field; and the sixth concludes by discussing the impact of sectoral ontologies on the performative capacity of promise. In doing so, this paper makes a significant contribution as the first social science analysis to critically engage with the emergent topic of substantial upscaling of tissue engineering across these domains.

## Technology Contexts: Cultured Blood

Red blood cell transfusions are widely used in modern medical care to overcome blood loss through trauma or surgery, or disorders that impair effective blood production, and currently rely on a supply of blood from human donors. Many richer nations have an established blood transfusion system but there remains many areas of the world without access to safe blood. However, donor numbers are decreasing and tighter controls to prevent Transfusion-Transmitted Infections (TTIs) are increasing the cost of transfusions. Many argue that within 20 years a crisis point will be reached in the ability of the transfusion services to supply the global blood demands of modern healthcare so an alternative method of blood transfusion is being sought.

Two existing technologies seek to provide an alternative to donated blood. ‘Synthetic’ blood equivalents are designed to replace the oxygen-carrying capacity of red blood cells but have generally proved unsuccessful with limited clinical use (Henkel-Honke and Oleck, 2007; Grethlein and Rajan, 2012). In contrast, reducing blood use through cell salvage and changing medical practices is proving more successful (Rees *et al*, 1996; Martyn *et al*, 2002).

Cultured blood technology follows a different path by working to manufacture various blood cell types – primarily red blood cells (for delivering oxygen) and plasma (for aiding clotting) – through tissue engineering. Red blood cells make up around 40–45% of the volume of human blood and are the main focus of the cultured blood research discussed here. The aim of research in this area is to produce an unlimited supply of laboratory-grown red blood cells, removing the need for blood donors, and creating an infection-free source. If O Rhesus negative blood is produced (the universal donor), then this could supply the majority of the population.

Research on cultured blood in its various forms has been overwhelmingly University based and dates back as far as 1993 (Rousseau *et al*, 2014; Sardonini and Wu, 1993; Koller *et al*, 1993; Palsson *et al*, 1993) with groups active internationally including in Spain (Ramos-Mejía *et al*, 2014), South Korea (Park *et al*, 2015), and the USA (Giani *et al*, 2016). A Japanese group working on clotting has used human pluripotent cells in steps to produce an “inexhaustible source of hPSC-derived platelets for clinical application” (Nakamura *et al*, 2014, p. 535). In France, a key group around Luc Donay produced red blood cells that matured inside mice (Neildez-Nguyen *et al*, 2002) and later developed a proof-of-principle protocol for using cultured blood in humans through which they injected 2 mL of cultured blood into a patient using the patient’s own cells as source material (Giarratana *et al*, 2011).

The group we have observed most closely is the UK-based Novosang project (previously BloodPharma project). This group originated in response to a call from the US Defense Advanced Research Projects Agency (DARPA), who wished to develop a method of producing blood on the battlefield. The team behind Novosang was unsuccessful in obtaining DARPA funding but continued to work on culturing red blood cells supported by UK funders. Although originally using human embryonic stem cells (hESCs) (Mountford, 2008; Mountford and Turner, 2011), they now use iPS cells from adult donors and anticipate that the final clinical product will be made entirely using adult cells. Using this, they have now developed “a clinical grade Good Manufacturing Practice protocol for using stem cell lines to manufacture red blood cells” (Wellcome Trust, 2017).

More recently, in May 2017, two papers from US laboratories published simultaneously in Nature reported the successful creation of hematopoietic stem cells that have the potential to produce all of the cell types found in blood, with one using induced pluripotency stem cells that developed human blood cells once injected into mice (Sugimura *et al*, 2017) and the other modifying existing mouse blood cells to produce hematopoietic stem cells (Lis *et al*, 2017) in work seen as early steps toward producing limitless supplies of blood (Johnston, 2017). The field has shown that laboratory-grown red blood cells are now possible but huge scale-up challenges exist to produce a standardized product sufficient to supply transfusion services internationally.

## Technology Contexts: Cultured Meat

Cultured meat technology seeks to produce muscle tissue that can be eaten as meat. The first attempts were around the time of the millennium through two similarly timed projects. The first, funded by NASA, sought to expand the muscle tissue of a goldfish to provide an increased mass of potentially edible muscle tissue as a way of exploring solutions to meat production during space travel (Benjaminson *et al*, 2002). Around the same time, the arts group SymbioticA, through their tissue culture and arts project, used biological tissue in their arts practice to grow small quantities of, firstly, fetal sheep tissue and, subsequently, frog tissue to produce muscle that could be consumed as meat (Zurr and Catts, 2003).

In 2005, a university-based research program began in the Netherlands, funded by the Dutch government department responsible for policy on agriculture and climate change. Spanning three universities, this group worked on basic cell culturing technique in Utrecht to derive a stable embryonic stem cell line from pig and in Eindhoven to explore how muscle tissue could be stimulated into increasing its growth levels through chemical and electrical stimulation. Other small laboratories emerged following this period in Sweden, the US, and Canada, all working on relatively small projects with relatively small numbers of people looking at the very early stages of developing small prototype tissue engineering muscle growth technologies and bioreactors. It was also during this period that the US-based third sector group New Harvest became visible in their work to support cultured meat through promotion, supporting networking, and raising philanthropic donations to fund early-stage research. In 2008, People for the Ethical Treatment of Animals (PETA) offered $1 m for the first group to produce a substantial quantity of chicken meat and by 2011 would fund Dr. Nick Genovese’s three year research project in the area. During this first decade of research, the preferred term for cultured meat of most scientists involved in the field was ‘in vitro meat.’

As the Dutch group’s project came to an end in 2010, one of the team, Professor Mark Post of Maastricht University, commented in a newspaper report (Specter, 2011) that, if he had sufficient funds, he could develop the world’s first laboratory-grown hamburger. He subsequently secured funding for this enterprise, leading to the most high-profile moment in cultured meat technology in 2013, when he and his team, funded by Google cofounder Sergey Brin, staged a press conference in which the world’s first cultured burger was cooked and eaten in front of the world’s media (O’Riordan *et al*, 2017). With the cultured burger came a shift from ‘in vitro meat’ to ‘cultured’ as the preferred term (Datar, 2016; Stephens and Lewis, 2017). Following the press conference, other groups in America, Israel, Russia, and the UK entered the field, and Post’s Maastricht laboratory remains active. By 2016, several start-up companies became active, most notably Mark Post’s “Mosa Meats,” Nick Genovese’s “Memphis Meats” (who showcased a proof-of-concept cultured meatball in 2016 and cultured chicken and duck meats in 2017), and New York-based “Modern Meadow,” who produced some “steak chips,” but now focus upon cultured leather. The move to leather saw the emergence of the new umbrella terms ‘cellular agriculture’ and the ‘post-animal bioeconomy’ to bracket together cultured animal products also including milk and egg white. Also at this time a second third sector group, the Good Food Institute, entered the space to promote and fund research, and introduced the new phrase “clean meat” to describe the technology. Most recently, in mid-2017, plant-based food start-up company Hampton Creek made the announcement that surprised some in the field that it aimed to sell a commercial cultured meat product by 2018. However, while there are an increasing number of groups involved, the field remains small with some arguing that the technology remains early stage.

## Promise, the Sociology of Expectations, and the Performative Turn in Economics

All technological innovations are embedded within sets of promissory narratives and future imaginaries (Bidault and Cummings, 1994; Lösch, 2006; Borup *et al*, 2006). These are used to set a framework of meaning for interpreting the work conducted so far and the work proposed for the future. These accounts are also important to enrolling financial, scientific, institutional, and public support for continued development. Such narratives are, inevitably, contested by detractors of the technology, or proponents of other technologies, who provide counter-narratives that articulate different frameworks of meaning and seek to align financial, scientific, and institutional resources in a different configuration. There is a growing literature documenting how scientists construct the promissory narratives and expectations around their own particular technologies (Hedgecoe, 2004) that has retained a sustained interest in biomedicine (Kitzinger and Williams, 2005; Pickersgill, 2011; Stephens and Dimond, 2015) including blood (Brown *et al*, 2006; Martin *et al*, 2008).

Promissory narratives are essential in mobilizing funding (Borup *et al*, 2006), bringing together interdisciplinary or multidisciplinary teams in the pursuit of a common goal (Lyall *et al*, 2011), and in justifying the funding of research that represents the best value for money. Through these mechanisms, and others, expectations offer the possibility of bringing into being the world they conjure through performative acts (Brown and Michael, 2003, 2014). The downside of promissory narratives is the susceptibility to path dependency and lock-in (Liebowitz and Margolis, 1995), where a change away from a nonoptimal technology may prove too costly.

Allied to the sociology of expectations literature are a set of authors working on what has been termed the ‘performative turn’ in economics (Muniesa, 2014), mostly focusing upon the capacity of models and broader imaginaries of market situations to offer the potential of bringing the markets they describe into reality. Callon (1998) argues that successful economic theories engage in world-making by pointing to a world and then bringing it into being. MacKenzie (2006) articulates a typology of performative mechanisms in financial markets to articulate how some equations – such as the Black–Scholes – lead people and markets to act in accordance with its predictions. Muniesa (2014) writes of the “provoked economy” through which economic ideas can realize (as in ‘make real’) the activities they describe. Importantly, the claim is that only a select few economic actions have the performative capacity to bring themselves into being. As MacKenzie (2006) notes, some have no impact on practice and, of those that do shape practice, some make markets less like those anticipated with only a subset molding markets into something like what they predict.

Callon (2007) argues that economic activities with the potential for performativity extend beyond formal economic modeling to include other technologies and forms of knowledge. As MacKenzie *et al* (2007) argue, performative acts can be as mundane as “suggestive vocabularies” (p. 6) for understanding future market contexts. Our work follows the move within this literature that steps away from financial markets to include diverse settings including fishery quotas (Holm and Nolde Nielsen, 2007), consumer preference testing (Muniesa, 2014), and computer system procurement (Pollock and Williams, 2010). In what follows, we explore the tentative performativities of cultured blood and cultured meat as novel and early-stage innovations to inspect the market situations both as they exist today and as they are imagined in the future.

## Methods

This paper draws upon two projects that were conducted independently. Emma King studied the UK cultured blood project from 2009 to 2013 conducting laboratory observation, documentary analysis, and outreach work alongside the cultured blood team (King, 2015). Emma and Catherine Lyall then conducted a study of public reactions to cultured blood, using focus groups and interviews (Lyall and King, 2016; King, 2017; King and Lyall, 2018). This focus group and interview research was led from Anonymous University and funded by the same Scottish Funding Council grant funding the scientific research. It explored the attitudes of publics and ‘experts’ in different fields (for example, ethicists, representatives of different religious groups, medical staff) toward the development and use of cultured red blood cells for transfusion. During this process, Emma and Catherine were embedded within the cultured blood team, attending regular meetings and conference calls in which the scientists spoke of their visions for cultured blood. This work found that there was overall positivity about the use of cultured red blood cells, with concerns focused on the potential problems of commercialization and future restriction on access to blood, rather than on concerns with the technology itself.

The second project, conducted by Neil Stephens, reports documentary analysis and an interview and observational study conducted with the scientists, funders, and proponents of cultured meat technology. This project commenced in 2009 with the bulk of the 42 interviews conducted between 2010 and 2013, and ongoing observational and documentary analysis continuing ever since. Neil has attended all international meetings of the field since 2010, including the press conference in which the cultured burger was tasted.

In 2013, the authors commenced discussions on developing comparative work across the independent projects leading to an extended period of dialogue. This included a joint meeting with the scientific team conducting the cultured blood research in 2014 during which the comparative work conducted to date was presented and discussed to generate further topics for consideration. Following this, the authors used their existing data sets to conduct a formal thematic comparison on eight themes: promissory narratives, future economic imaginaries, anticipated regulations, institutional forms, laboratory work, ontological status, socio-ethical debate, and the cultural and media landscape. The comparison of the first six of these themes constitutes the substantive focus of this paper, while the comparison of the remaining two informs our analysis but features less prominently. The dialogic comparative work necessitated a further period of data collection during 2015–2017 through ongoing contact with the practitioner community and an updated literature review on recent developments in cultured meat and extending the international focus of our work on cultured blood. The projects have been considered and approved independently by the Research and Ethics Committees at the University of Edinburgh and Cardiff University. Collectively, the two studies on cultured blood, the extended study of cultured meat, and the sustained subsequent cross-analysis of both constitute a robust and significant dataset from which our argument is drawn.

## The Promissory Narratives of Culturing Blood and Meat

We now identify eight promissory narratives evident in the scientists’ accounts for both cultured blood and cultured meat. These classifications allow us to highlight shared narratives, while also making explicit how they are differently constructed in the biomedical and agri-food sectors.

### The achievability narrative

Both cultured meat and cultured blood suggest that significant upscale of stem cell tissue engineering is possible. Currently, blood use in the UK alone stands at around 2.2 million units a year, with each bag of blood containing 2.5 trillion cells (Mountford and Turner, 2011). Estimating global blood markets is difficult because many countries with developing healthcare systems currently lack a blood transfusion service that can provide comparative figures. Nevertheless, replacing just UK blood donation with a tissue engineering-based system represents significant upscale compared with any existing stem cell technology today. Yet, blood consumption is minute compared to global meat consumption, estimated to be 278,863 thousand tonnes of tissue in 2009 alone (Henchion *et al*, 2014), and rising. The boldness of the promissory narrative that suggests the possibility of these quantities is captured in a comment by a plenary speaker and bioengineer at the 2015 Cultured Meat Symposium, who claimed that currently there is not enough stainless steel in the world to build the bioreactors required. For both cultured blood and cultured meat, the narrative of achievability is bold and far beyond that of any other current stem cell technology. Bioengineers Chris Hewitt and Qasin Rafiq calculate that, if the entire world’s bioreactor capacity of around one million liters were turned over to culturing meat, then it would only be capable of feeding 400,000 people, suggesting that significant investment in building bioreactors would be needed (Hodson, 2015).

### The crisis narrative

Both technologies are linked to a crisis narrative about pessimistic futures that should be guarded against. For cultured blood, the crisis point focuses upon the pressure on the blood transfusion services over the coming decades. Falling donor numbers, growing and aging populations, and the increasing costs of testing and processing are core justifications for pursuing alternatives to the current system. In addition, there is a global narrative around supplying blood to a wider population, as we pick up in the ‘global poverty narrative’ below.

In cultured meat, the crisis is quite different; it is a crisis of the environment, land use, and population. Proponents of cultured meat cite the UN Agricultural Group’s report *Livestock’s Long Shadow* (Steinfeld, 2006) about the impact of meat production on deforestation, greenhouse gas emissions, and environmental degradation. This has been supported by a widely cited life-cycle analysis of a hypothetical system compared with existing livestock practices that suggested greenhouse gas emissions could be 78–96% lower and water use could be 82–96% lower than meat (Tuomisto and Teixeira de Mattos, 2011). This is distinctive among tissue engineering technologies as it invokes an environmental promissory narrative not found anywhere else within the field.

### The minimized infection narrative

Both cultured blood and cultured meat proponents suggest that their cultured tissue will be less infected than the tissue they seek to replace. The main concerns are both diseases found in tissue and the antibiotics used to control these diseases. In the cultured meat case, the focus is animal-borne disease based on precedents such as CJD and bird flu found in farming environments. Since a cultured meat system uses significantly fewer animals and the tissue eaten is cultured without a living body, the incidence of disease and antibiotic use is thought to be significantly reduced.

In cultured blood, the focus is Transfusion-Transmitted Infections (TTIs), including Hepatitis C and HIV, transmitted through blood transfusion. Stricter controls are leading to lower eligible donor numbers and increased processing costs for donated blood, in addition to preparing for future unknown TTIs. The issue of antibiotics is less pronounced in cultured blood than it is in cultured meat, as many countries place restrictions on donors who have recently used antibiotics.

### The global poverty narrative

Both cultured blood and cultured meat are associated with global poverty. For cultured meat, this relates to global food poverty and justice issues around hunger and malnutrition. With cultured blood, this is primarily articulated as being about access to safe blood in developing nations, especially areas with fast developing healthcare systems but high levels of endemic infections. However, it is noticeable that in both cultured meat and cultured blood there is uncertainty within the respective research communities about the deliverability on these issues. In the cultured meat case, the narrative is frequently softened with accounts about the impact of geopolitical issues in global food distribution as key issues beyond the sheer quantity of food available. In the cultured blood case, questions are raised over the capacity to produce and store cultured blood effectively in less wealthy nations given the likely requirement for significant refrigeration facilities. In areas with poor healthcare provision, access to medical care is the limiting factor for patient survival, rather than the amount of blood available.

### The enhancement narrative

Both technologies are associated with narratives around improving upon current blood and meat. In the context of cultured blood, the main anticipated added value over donated blood is (i) the potential to produce generic O negative blood type that can be used by almost anyone; (ii) the potential to create an improved therapy for multiply transfused patients (due to only transfusing ‘young’ red blood cells); and (iii) producing rare blood groups for patients who cannot currently be transfused. For cultured meat, the enhancements are seen in (i) healthier meats, with either additional nutrition engineered in, or fats and disease-provoking amino acids engineered out; and (ii) innovative meats that taste better, or look better, and take on new forms, and to a lesser extent meats from animals such as lions that are insufficiently docile to farm easily in industrial contexts.

### The ‘biological source’ narrative

Embedded within the narratives of both technologies is a focus upon the living beings that provide standard blood and meat; human for blood and industrialized animals for meat. However, the issues are configured very differently. Human blood donors are seen as insufficient in numbers to provide the quantity of blood needed. Livestock animals are seen as having insufficient space and resources on the planet’s surface for them to supply the meat we need. Livestock animals are also configured as ethically sensitive beings that need protection from slaughter, to produce a ‘meat without murder.’ Both cultured blood and cultured meat are said to address this by requiring significantly fewer living beings to deliver the required tissue. This noted, both cultured blood and cultured meat still require some biological source from tissue donors, raising issues about the provenance of this initial tissue.

### The military/astronaut narrative

Early interest in both technologies came from the large American governmental agencies. For cultured blood, this was DARPA’s interest in the military applications of a blood that could be used easily and safely on a battlefield. For cultured meat, it was NASA’s interest in providing meat during long-term space travel (Benjaminson *et al*, 2002). In both cases, the users are professionals working under the extreme conditions of war or space travel in the national scientific and political interest. The astronaut narrative subsequently became played down by the field for over a decade as the other narratives became established and dominant, until a group at North Carolina State University started referencing space travel in the mid-2010s for their work on culturing turkey muscle, and New Harvest attended a NASA workshop on innovative food for space travel in 2017.

### The financial narrative

Both cultured blood and cultured meat have been associated with the capacity to make money and develop a high-skill knowledge economy, particularly in the case of cultured meat, where tissue engineering expertise is positioned as a higher quality skill set than many other roles in current livestock agriculture. The form that this economic component is anticipated to take is the focus of our next analytical section.

## Markets and Anticipated Futures

Like any technology, the scientists active in both cultured blood and cultured meat operate with a set of anticipated futures. While the futures of each technology remain contested within their scientific communities, there are clear themes developing that speak to the anticipated consumer, the anticipated market, and the anticipated regulatory pathway. These anticipated futures shape current practices through an anticipation of potential opportunities and challenges. The literature on the performative turn in economics is therefore relevant even for these early-stage biological projects. In this section, we explore three issues – consumer publics, market contexts, and regulatory pathways – but first we reflect upon the current market situations of each technology.

### Current market situation

Cultured blood and cultured meat are both early-stage technologies, and their current market situations reflect that. That said, important differences remain between the two in terms of the institutional forms that their funding streams take. As noted above, the UK cultured blood team began in response to a call from DARPA but they subsequently attracted substantial mainstream funding through a biomedical – as opposed to defence – route, from the Wellcome Trust, the world’s largest medical research funding charity. The Scottish Funding Council has also been a major funder of the cultured blood project along with blood transfusion services across the UK. Funders of the Japanese cultured blood work included the government via the Ministry of Education, Culture, Sports, Science and Technology (MEXT) and the Ministry of Health Labor and Welfare, and funders of the French team include the Association pour la Recherche en Transfusion (Research and Transfusion Association – a charity funding projects around blood transfusion technologies since 1968), a national public institution the Etablissement Français des Greffes (the French Graft Establishment), and the Assistance Publique-Hôpitaux de Paris (the Paris public hospital system). The theme of governmental and established charity funders continues in the 2017 US papers with Sugimura *et al* (2017) drawing support from National Institutes of Health, the Crohn’s and Colitis Foundation of America, and the Doris Duke Medical Foundation, while Lis *et al* (2017) counts the National Institutes of Health, the Leukemia & Lymphoma Society, and the Qatar National Priorities Research Program, among its funders. Funding from these respected institutions lends credibility to the work.

The cultured meat funding context contrasts starkly to that of cultured blood. The cultured meat field is characterized by an unusual constellation of funding bodies and small supporting groups. We have already noted the early work of NASA and bio-arts group SymbioticA. Other early work was funded by the pro-cultured meat third sector group New Harvest, who continue to provide funds and promotional support through PhD studentships. The 2005 Dutch project was funded by a governmental body interested in the environment. People for the Ethical treatment of Animals (PETA) also funded a 3-year postdoctoral project in America. University-based laboratories in Canada and Sweden have been largely self-funding, sometimes relying upon Masters students’ projects to keep the research area operative. As noted above, Mark Post’s laboratory was funded by Google’s Sergey Brin after a media campaign.

There is also a set of small start-up companies active in the area. Further extending the role of IT entrepreneurs in cultured meat, the start-up company Modern Meadow in America has attracted venture capital and funding streams such as the Breakout Labs scheme, supported by PayPal founder Peter Theil. Most recently, start-up Memphis Meats announced in August 2017 that they had received funding of $17 million from investors including Bill Gates, Richard Branson, and global food corporation Cargill. Mark Post has himself started a company called Mosa Meat. Third sector group the Good Food Institute also funnel philanthropic derived funds to start-ups via an allied private venture capital fund New Crop Capital, which has supported Memphis Meats among others. The Israel-based start-up SuperMeat raised $215,380 through crowdfunding website Indiegogo. The USbased companies often have links to the Singularity University or Indiebio, both Californiabased tech incubators focused on disruptive technologies.

In recent years, cultured meat has attracted the support of some within the Effective Altruism movement. The central tenets of this utilitarian ethics and impact assessment-based approach to philanthropy is articulated in Macaskill’s (2015) *Doing Good Better* and Singer’s (2015) *The Most Good You Can Do*. The Effective Altruism (2017) website advocates cultured meat through reference to an Open Philanthropy Project (2016) investigation and in 2017 gave a direct donation to New Harvest of $10,000. Another group focused upon animal issues, Animal Charity Evaluators (2016), rates New Harvest as a “Standout Charity” and Good Food Institute as a “Top Charity” suitable for philanthropic donations. In contrast, there is no equivalent Effective Altruism support for cultured blood, with the only activity in the area working to promote standard blood donation (Aceso Under Glass, 2015).

These funding differences speak to the distinctive space occupied by cultured meat. None of the above-listed funders – arts groups, newly established third sector organizations, IT entrepreneurs, vegan venture capitalist companies, crowdfunding websites, or disruptive philanthropic platforms – are typical funders of tissue engineering research or are known to have any role in supporting cultured blood. As we argue, these current market situations both inform and are informed by anticipated future economic contexts. We now articulate the anticipated publics, markets, and regulatory regimes for both technologies.

### Anticipated consumer publics

Proponents of cultured blood imagine it operating in the space currently occupied by blood donation systems, namely medical settings within public and private healthcare. Proponents of cultured blood imagine different contexts of use within advanced economies with sophisticated and well-funded healthcare systems through to poorer nations in the world, where the support infrastructures, economic realities, and the health status of the patient could be configured differently. Early adopters are anticipated to be patients with disorders preventing effective blood production such as thalassemia, sickle cell, or some types of cancer. As noted, there is also the potential for military uses of cultured blood technology.

In cultured blood, the amount of choice that the consumer will have is unclear and could depend upon the national regulation within each jurisdiction. In the UK with its National Health Service for example, a wider decision to switch to cultured blood may leave individual patients with very little choice over the blood that they receive. Given the use of blood in emergency medicine, it is also likely that many people may be transfused in situations in which they were unable to give consent. Public concerns about commercialization and the potential role of private companies in the future replacing the current blood donation system were one of the overarching findings from the study of public attitudes to cultured blood (Lyall and King, 2016; King and Lyall, 2018).

There has been little work to date concerned with producing empirically based projections on consumer publics’ attitudes to alternative blood products. The project reported here by Emma and Catherine (Lyall and King, 2016; King and Lyall, 2018) was the first project to look specifically at public attitudes to cultured blood, although work is now being done by a team based at the University of Bristol.^2^ The only other study in the area is the EuroBloodSubstitutes project from the mid-2000s with participants from the UK and Holland that included donor blood, GM blood, chemical blood, and bovine blood but not cultured blood (Ferguson *et al*, 2008).

Cultured meat anticipates a more novel type of consumer public, embedded clearly within food consumption in sites of the home and restaurant. The anticipated cultured meat consumer publics are ethically informed, politically active citizens, who seek to remove ethically and environmentally problematic elements of their consumption without becoming vegetarian or vegan (Stephens, 2013; O’Riordan *et al*, 2017). The consumer’s capacity for choice is central to how they are anticipated: they choose cultured meat to contribute to a better world without experiencing a change in their experience of eating.

In contrast to cultured blood, there are an increasing number of projections for public’s perceptions of cultured meat. These range from academic studies using diverse methodologies to journalistic online surveys that in turn are quoted in other media contexts. Academic cultural studies’ analyses of social media responses include Laestadius (2015), Laestadius and Caldwell (2015), and O’Riordan *et al* (2017). Published focus group research has been conducted in the Netherlands (van der Weele and Driessen, 2013), Ireland (Department of Agriculture, Food and the Marine, 2013), the USA (Hart Research Associates, 2017), Finland (Vinnari and Tapio, 2009), the UK (Bows *et al*, 2012; O’Keefe *et al*, 2016), and a comparative study of Belgium, the UK, and Portugal (Marcu *et al*, 2015; Verbeke *et al*, 2015), and we know of other as-yet unpublished academic focus group work conducted in Spain and the UK. There has also been a survey of 1,800 people (Hocquette *et al*, 2015), another of 673 people in the USA (Wilks and Phillips, 2017), and an experimental psychology project also conducted in The Netherlands (Bekker *et al*, 2017). In addition to this, the Good Food Institute have produced work criticizing existing projections on consumer responses claiming that flawed methodology (for example, using negative terminology or underspecifying potential benefits) led them to exaggerate negative responses (Friedrich, 2016), and Effective Altruism group Animal Charity Evaluators (2017) conducted a randomized trial of whether the term ‘cultured’ or ‘clean’ meat most appeals to consumers. Collectively, these studies report diverse opinions from the strongly supportive to the strongly resistant, while also documenting sets of ambiguities and uncertainties about the personal, societal, and political context of the technology.

### Anticipated market context

Both technologies are anticipated to initially enter a mixed economy, where traditional blood and traditional meat are still available as competitor products. The significant quantities of tissue anticipated in replacing all the donated blood in the world, or all the meat consumed in the world, mean that swift and absolute replacement of competitor technologies anticipates a level of upscale far beyond what is initially likely.

In terms of cultured blood, in advanced economies, the scientists want hospital administrations to pay for the blood. However, in the initial instance, due to the expense of clinical trials, it is more likely that the involvement of pharmaceutical companies or venture capital may be required in order to produce a marketable tissue-engineered product.

In the UK, blood is seen as a ‘free commodity.’ However, the current ‘cost’ of blood in the UK is around £120 per unit^3^ (approximately $150); however, this could rise to £600–800 ($775–1000) if the full cost of infrastructure is taken into account (Varney and Guest, 2003). Rousseau *et al* (2014) reference Timmins and Nielsen *et al* (2009) and Zeuner *et al* (2012) to suggest that, at current technology, one unit of blood could cost $8,000–15,000 to produce, which they compare to the typical $225 current hospital cost for a unit of leukocyte-reduced red blood cells. However, there may be instances where a higher price could be justified; for example, cultured blood use by thalassemic patients could result in fewer transfusions, reduced side effects, and a longer life span and may, therefore, justify a higher cost. Taking a similar line, Rousseau *et al* (2014) suggest that the current cost per unit for allo-immunized patients is $700–1200, and predict that this cost would also be met by health insurance companies in critical situations. This given, these prices are clearly below the current likely costs with Rousseau *et al* (2014) identifying a cultured blood cost of $3,000 per unit as the appropriate target which remains higher than even rare blood types at current prices.

The anticipated economics of cultured meat are quite distinct, once again in the world of agri-food as opposed to healthcare. The economic context of meat production differs significantly from traditional healthcare and therapeutic product markets and meat prices remain significantly lower than those for biomedical technologies. As opposed to $225-per-unit blood supplies, cultured meat proponents compare their tissue engineering technology to burgers, sausages, and beef steaks found in supermarkets.

The vastly different pricing strategies found in the healthcare and food sectors raise significant challenges for the cultured meat community. Cultured meat proponents remain open to a diversity of commercialization routes for their technology, including like-for-like replacements of existing processed meat products, or locally produced artisan cultured meat facilities in each town and city able to support one (van der Weele and Tramper, 2014). Cultured meat products are anticipated to initially be high-end, ethically desirable products, with a slightly higher price point than standard meat products, comparable with organic or free range meat although, over time, scientists hope that the price could fall in line with all meat products. This given, an alternative route remains of cultured meat not only entering a mixed economy but also entering mixed products of, for example, sausages combining both whole animal and cultured meat product. This mixing approach reconfigures the promissory narratives, with those around environmental crisis, infection, and animal welfare weakened when mixed with livestock meat, although resultant financial opportunities may be increased.

Anticipated regulations: Both technologies remain at an early stage of development. However, future regulatory pathways are still anticipated by the scientists involved and form an important component of their market context. In both cultured blood and cultured meat, there remain uncertainty about exactly what form this regulatory pathway would take, although the clear distinction between biomedical and agri-food sectors is recognized. During the UK Novosang project, the human embryonic stem cells used in the early development of cultured blood required licensing from the Human Fertilisation and Embryology Authority (HFEA). Whether embryonic or adult cells are used, it is expected that EU clinical cultured blood products would be regulated by the national competent authority of the European Medicines Agency as an Advanced Therapy Medicinal Product, with equivalent pathways followed in other jurisdictions (Mittra *et al*, 2015).

Anticipating the regulatory pathway in cultured meat involves additional uncertainties and creativity in practice for the scientists involved. As in the cultured blood case, cultured meat scientists are primarily biomedical. They are primarily individuals with expertise built in the healthcare industry around cell culturing, tissue engineering, and bioreactor design. For these individuals, the move into food regulation presents a new and unfamiliar terrain that they seek to learn about through strategic alliances with consultancy professionals and food manufacturing organizations. Attempting to address this, third sector organization the Good Food Institute have staff working on articulating and assessing policy pathways in different countries. Two existing analyses produced by lawyers of potential regulatory pathways in the EU (Petetin, 2014) and the USA (Schneider, 2013) concluded that current regulation was not suitable to cope with the definitional challenges of cultured meat, and that technical uncertainties in what form the technology may take mean the regulatory trajectory is inherently ambiguous.

## Discussion: Promissory Narratives, Anticipated Markets, and Sectoral Ontologies

This paper argues that cultured blood and cultured meat share eight promissory themes, although the form these take varies with the specifics of a biomedical/blood and an agri-food/meat context. While both promise the achievability of upscale, cultured meat implies a higher tissue yield to replace existing systems (in the UK over 5,000,000 tonnes of meat per year compared to around 176 tonnes of blood), and deliver it at a much lower price point (around £2 per burger compared with £100 per bag of blood). Both technologies are embedded within a crisis narrative based upon shortcomings in current provision, be that around pressures on blood transfusion or pressures on the environment. This has some overlap with the shared minimized infection narrative in which the control of sterile production facilities promises to minimize the disease and antibiotic risk in the tissue we eat or have transfused into our bodies. Further health benefits are claimed with an enhancement narrative through engineering only generic O negative blood type, or engineering in additional nutritional benefits. The biological source narrative relates to the beings from which tissue is traditionally taken, be that insufficiently numbered blood donors or industrialized and poorly treated livestock. The military/astronaut narrative captures the role of large governmental agencies keen to use cultured technologies to benefit their employees in extreme conditions. Finally, both promise financial reward, and we have detailed the current and anticipated market contexts through which this might occur.

Promissory narratives are important constitutive components of both cultured blood and cultured meat, giving direction, structure, and enrolling support. However, promissory narratives are also malleable and can be reconfigured over time. This is evident in both of our case studies. The UK cultured blood group formalized their actions around a defence-orientated funding call before consolidating their research around the biomedical narrative of the Wellcome Trust and blood services. In cultured meat, we saw initial work entirely focused around delivering food for space travel, followed by a complete reconfiguration around a new set of terrestrial promises, before space once again entered the scene as a new group voiced their anticipated goals in 2016 and New Harvest attended the NASA event in 2017.

The key commonality between cultured blood and cultured meat is the shared focus on a step change increase in the upscale of stem cell-based tissue engineering, while the key difference is the context of marketization in biomedical or agri-food sectors. We must note that, in these differing contexts of marketization, cultured meat has had to make a switch that cultured blood has not. Cultured meat technology draws upon biomedical innovation that is being applied in the agri-food sector. This switch from biomedicine to food has raised challenges for the scientists involved in terms of the status and institutional support afforded their technology. The issue of status operates at a profound level, beyond the credibility of whether cultured meat can deliver its promises, into questions of what cultured meat actually *is*. Capturing this, Stephens (2010, p. 400) described cultured meat as an “as-yet undefined ontological object” to articulate potential ambiguities about whether tissue-engineered muscle really can be understood as meat, or as a meat alternative, or simply not as food at all.^4^ The shift in sector is key to amplifying this ambiguity: while tissue engineering in biomedical contexts is an accepted category of practice – it has generally become ‘normal’ – and makes little challenge to existing expectations around how biology is done. That is not to say there is no ontological ambiguity around cultured blood. The use of red blood cells themselves does raise questions about how this particular product is perceived and categorized, sitting between ‘real’ donated red blood cells and a synthetic pharmaceutical (King, 2017; King and Lyall, 2018), but this ambiguity is less dramatic than meat because tissue engineering has become established within biomedicine. Shifting tissue engineering into the agri-food sector engenders ambiguity, as it clashes with well-established understandings of what meat is and how it should be produced. In other work, Stephens (2013) argues that promissory narratives are used in an attempt to resolve the ontological ambiguity of cultured meat, and that the cultured burger press conference was a key moment in stabilizing both its meanings and the potential consumer publics that will buy it (O’Riordan *et al*, 2017; Stephens and Ruivenkamp, 2016). Here we close by exploring the sectoral ontologies of cultured blood and cultured meat and the impact this has on the performative potential of promissory narratives to bring their promises into being.

By sectoral ontologies, we mean the categorizations and methods of organization and sense-making typical of the economic and scientific sectors of biomedicine and agri-food. The origin and application narratives of cultured blood exist within a single sectoral ontology, while the origin and application narratives of cultured meat shift between the sectoral ontologies of biomedicine and agri-food. This, we argue, informs the current market situation of both technologies. The cultured meat field is characterized by an unusual constellation of funding bodies and small groups that exemplify the ontological ambiguity over what cultured meat is. In the absence of a clear definition of what cultured meat is, there is also an absence of established institutions to support it. Ontological and institutional alignment supports the performativity of promises and economic activities.

Cultured blood – being unambiguously biomedical – benefits from aligned sectoral ontology in origin and application, as evidenced in funding from prestigious organizations such as the National Institutes of Health, the Wellcome Trust, and Etablissement Français des Greffes. The economic activities of cultured blood groups exist within a universitybased, publicly and established charity-funded biomedical context, and the individuals involved are overwhelmingly experts in various forms of biomedical engineering. The institutional and the ontological are aligned, facilitating economic performativities.

Cultured meat, conversely, exists in a less mainstream funding context. Developers of cultured meat must work to assert these alignments in order to benefit from them. Groups such as New Harvest and the Good Food Institute actively seek opportunities to connect finance flows with the distinct and novel promissory and market context of cultured meat. Their suggestive vocabularies therefore need to work harder than those of cultured blood to provoke the world they suggest into being. Furthermore, the majority of scientists in cultured meat are trained in biomedicine, and those based in universities often run biomedical research in parallel to their agri-food work. These biomedical researchers work in conjunction with a smaller number of food and meat professionals and scientists, and through graduate programs such as that supported by New Harvest we are only now seeing an early wave of researchers trained specifically in this field. This hybridity is illustrative of the ways in which cultured meat does not easily fit the typical structures of neither the agrifood nor the biomedical sector, as additional sense-making work is required to form meaningful alignments. The ontological ambiguity of cultured meat frames these institutional and sectorial hybridities and thus inhibits the performative character of their promises.

This carries forth into anticipated consumer publics, marketization, and regulatory pathways. For cultured blood, consumer publics, pathways through regulation, and the mode of marketization are relatively clear and resilient: in pre-Brexit UK, this is European Medicines Agency approval and NHS service provision. For cultured meat, a regulatory pathway and the attendant methods of scrutiny require development, a marketing model needs advancing, and consumer publics are still to be enrolled. The clash of sectoral ontologies brings with it clear practical challenges.

While promissory pathways can be essential in mobilizing funding and establishing development trajectories, imaginaries can also fail to perform markets into being. Questions still remain over the technical feasibility of both cultured blood and cultured meat as an upscaled industrial reality. These early-stage technologies retain early-stage imaginaries of their future pathway to commercialization, and potential barriers to entry may remain underestimated (cf. Gunnarsdottir *et al*, 2015 for a cultured meat example). What is clear is that cultured blood has several advantages over cultured meat: the long-term tissue yield goal is lower, the likely competitive price point is higher, and the stability of its sectoral ontology furnishes it with an institutional context that better affords the performative capacity of promise. In contrast, cultured meat proponents could equally point to production advantages in that the tissue need not still be living at the point of use, and that food regulatory hurdles are likely to be lower than their biomedical equivalents. However these futures are played out, blood and meat are currently the most ambitious sites of promised tissue engineering upscale. Their long-term commercial viability remains indeterminate but their current contexts reveal pertinent insight into the role of promise, performance, and sectoral ontology. In documenting these, we have begun the important work of analyzing significant upscale as a distinct set of practices requiring social science assessment and have pointed to the need for subsequent studies exploring upscale in other contexts.

## Footnotes

1 Blood figure provided by the UK cultured blood Novosang project (http://novosang.co.uk), meat figure calculated by authors based upon Food and Agricultural Organization of the United Nations Statistics Division data for 2011 (FAOSTAT, 2016).

2
http://www.hra.nhs.uk/news/research-summaries/public-and-patient-views-of-laboratory-grown-red-blood-cells/.

3 In England, the standard tariff for blood supplied by NHSBT is set yearly, with hospitals paying for each unit of blood used (NHS England and Monitor, 2016). In 2014/15, the cost was £121.85 per unit; in 2015/16, it was £120 per unit. http://www.nhsbt.nhs.uk/news-and-media/news-articles/news_2015_07_10.asp. In Scotland, the SNBTS and the blood it produces are centrally funded from the core NHS budget, but the cost would be similar.

4 See also Chiles (2013), Kramer (2015), Jönsson (2016) and Stephens and Ruivenkamp (2016) on the relation between promise and ambiguity for cultured meat.
